# Rapid Prototyping of Inertial MEMS Devices through Structural Optimization

**DOI:** 10.3390/s21155064

**Published:** 2021-07-26

**Authors:** Daniele Giannini, Giacomo Bonaccorsi, Francesco Braghin

**Affiliations:** 1 Department of Mechanical Engineering, Politecnico di Milano, Via G. La Masa 1, 20156 Milano, Italy; giacomo.bonaccorsi@polimi.it (G.B.); francesco.braghin@polimi.it (F.B.); 2 Department of Civil Engineering—Structural Mechanics Section, KU Leuven, Kasteelpark Arenberg 40, 3001 Leuven, Belgium

**Keywords:** structural optimization, inertial MEMS, MEMS gyroscopes, MEMS, beating-heart gyroscope, device-level MEMS schematization

## Abstract

In this paper, we propose a novel design and optimization environment for inertial MEMS devices based on a computationally efficient schematization of the structure at the a device level. This allows us to obtain a flexible and efficient design optimization tool, particularly useful for rapid device prototyping. The presented design environment—*feMEMSlite*—handles the parametric generation of the structure geometry, the simulation of its dynamic behavior, and a gradient-based layout optimization. The methodology addresses the design of general inertial MEMS devices employing suspended proof masses, in which the focus is typically on the dynamics associated with the first vibration modes. In particular, the proposed design tool is tested on a triaxial beating-heart MEMS gyroscope, an industrially relevant and adequately complex example. The sensor layout is schematized by treating the proof masses as rigid bodies, discretizing flexural springs by Timoshenko beam finite elements, and accounting for electrostatic softening effects by additional negative spring constants. The MEMS device is then optimized according to two possible formulations of the optimization problem, including typical design requirements from the MEMS industry, with particular focus on the tuning of the structural eigenfrequencies and on the maximization of the response to external angular rates. The validity of the proposed approach is then assessed through a comparison with full FEM schematizations: rapidly prototyped layouts at the device level show a good performance when simulated with more complex models and therefore require only minor adjustments to accomplish the subsequent physical-level design.

## 1. Introduction

The popularity of inertial MEMS (Micro Electro-Mechanical Systems) sensors, namely accelerometers and gyroscopes, has grown continuously in recent decades. These devices, initially developed for military and space use, are now found in several automotive and consumer electronics applications and are employed in a large number of everyday products [1].

Both MEMS accelerometers and gyroscopes employ internal suspended-proof masses to detect linear accelerations and angular velocities, respectively. In particular, the most relevant inertial MEMS devices in terms of industrial diffusion and complexity in the internal microstructure are probably Amplitude Modulated (AM) Coriolis Vibrating Gyroscopes (CVG): these employ vibrating proof masses and leverage coupling effects between the structural resonant modes, which, in the presence of external angular rates, are caused by Coriolis forces [1,2,3]. This operating principle has been used in several MEMS gyroscope implementations, whose layout complexity has increased during the recent decades [3] due to continuously increasing market requirements on device miniaturization and performance. In this regard, the most challenging layouts are indeed multi-axis ones: by employing several proof masses, suspended and coupled by different springs, such devices allow to detect external angular rates along multiple Cartesian axes with one single complex structure [4,5,6,7,8].

The design of complex MEMS gyroscopes and inertial MEMS devices in general is not an easy task. As explained in [9], the usual design approach is split into four hierarchical levels (System, Device, Physical, and Process), each characterized by an increasing level of complexity and detail. The *system* level, at the top, focuses on block-diagram and lumped parameter descriptions, leading to a coupled set of ordinary differential equations (ODEs) to describe the dynamics of the system. At the *device* level, macro-models or reduced-order models are usually employed to capture the essential behavior of the system components while having a compatible description with the system level. At the *physical* level, the device is studied as a three-dimensional continuum, whose behavior is described by partial differential equations (PDEs) and modeled through finite-element, boundary-element, or finite-difference methods. Finally, the *process* level, at the bottom, is where the detailed design of the process sequence and the photomask for manufacturing is performed. Here, the modeling is a highly sophisticated numerical activity for which a number of specific commercial tools have been developed.

At each of the presented levels of detail, the design of inertial MEMS devices is usually trial and error and therefore largely depends on the expertise of the design engineer. MEMS devices are typically designed starting from simple mechanical elements (e.g., simple or folded beams, proof masses, and forcing and sensing electrodes) that are then combined through scaling laws, parametric studies, and engineering insight. In general, finding a satisfying combination that complies with technological, application, and performance requirements involves a highly costly and time-consuming design process. Many iterations are in fact manually performed to adjust the local geometries of the device and to validate the expected improvements via numerical simulations and experimental studies [10,11]. In order to overcome the limitations of the traditional design process, there is therefore a growing interest in the development of automatic design techniques for MEMS devices, especially by relying on structural optimization approaches.

An interesting strategy to speed up the design of MEMS devices is the use of parametric macro-models, implemented in several tools developed in academia, such as NODAS from Carnegie Mellon University [12] or SUGAR from UC Berkeley [13], as well as in commercial tools such as Coventor MEMS+ [14]. In these cases, the MEMS layout description relies on the combination of different building blocks (beams, masses, anchors, electrodes, etc.), whose basic geometrical properties are parametrized. This is particularly suitable when performing parametric studies or sweeps in the geometrical parameters and can be seen as a first preliminary step towards automatic design by numerical optimization techniques.

The optimization technique with the biggest potentialities of application in the inertial MEMS industry is structural size optimization: in this approach, the dimensions and positions of the mechanical elements composing the structure are parametrized by a set of design variables, which are then optimized by employing usual methods for numerical optimization, such as gradient-based [15] or stochastic/evolutionary techniques [16,17]. Size optimization has been applied to the design of several MEMS devices, e.g., to extend the operational frequency range in piezoelectric MEMS energy harvesters [18], to tailor mechanical nonlinearities through non-uniform beam profiles [19], or to improve temperature stability of tuning fork resonators through slots in the resonator beams [20].

Topology optimization techniques that do not require any initial layout parametrization are also gaining traction in the field, mainly due to their ability of exploring non-intuitive design spaces [21]. This approach has recently been applied to a broad range of relevant MEMS resonator design cases [22,23,24,25,26], including MEMS gyroscopes [27]. However, topology optimization is still not mature enough to forecast its integration in the MEMS industry in the very near future.

In the case of MEMS gyroscopes, one of the most challenging design cases is represented by triaxial layouts, whose complex internal microstructure has to be carefully designed to match target resonant frequencies while simultaneously maximizing the sensor response to the three Cartesian components of the external angular rate. The structural size optimization of triaxial gyroscopes was first addressed in [16,28], where the focus was on the optimization of the flexible elements, while the layout of electrodes and proof masses were decided a priori. The optimization relied on a particle swarm algorithm and aimed at tuning the structural eigenfrequencies, which were estimated through simple analytical models. The approximation errors associated with such simplified computations were then manually compensated for after the optimization, with the aid of commercial FEM softwares.

In a recent publication [29], the authors proposed a novel automatic design environment for inertial MEMS structures (*feMEMS*) that allows the layout to be optimized directly at the physical level. The layout of the MEMS device was parametrized and automatically generated, while the physical-level simulations of its dynamic behavior were performed using 3D finite elements, and the simulation tool was coupled with a gradient based optimization algorithm to iteratively update the design based on user-defined requirements. In addition, computational costs were reduced through the use of substructuring techniques for speeding up simulations and adjoint methods for analytical sensitivity computations. However, this approach requires the design engineer to perform analytical derivations for sensitivity analysis whenever new objective functions or constraints are considered in the optimization problem and to introduce the related low-level code modifications in the optimization tool.

The aim of the present work is to show how *feMEMSlite*, an automatic design tool for rapid prototyping, can be used to optimize MEMS layouts in a flexible and computationally efficient way. The proposed approach employs a computationally cheap, device-level schematization of the MEMS structure: proof masses are treated as rigid bodies, springs are discretized by Timoshenko beam finite elements (as in, e.g., [30]), and electrostatic softening effects are accounted for by adding equivalent negative spring constants. The related fast simulations allow finite difference schemes to be employed for derivatives computation without the need of adjoint sensitivity analyses: this increases the flexibility of the tool, which does not require any extra work for computing analytical derivatives and implementing low-level code adaptations. In particular, *feMEMSlite* is presented by focusing on the reference design case of a triaxial beating heart MEMS gyroscope layout, which is optimized to guarantee target natural frequencies and to maximize its response to the external angular rate. The choice of the same design case as in [29] will allow for a straightforward comparison between *feMEMS* and *feMEMSlite* in order to underline the differences between optimizing the structure at physical level and at device level, in terms of computational costs and model accuracy.

The paper is organized as follows. A general overview of *feMEMSlite* is described in Section 2, presenting the iterative scheme performed by the design tool to optimize the MEMS layout. Section 3 presents the considered design case of a triaxial beating heart MEMS gyroscope, summarizing its operating principle and main working requirements and introducing the related parametrization with design variables. Section 4 explains the device-level model used to study the MEMS dynamics in terms of mechanical and linear electrostatic effects. The considered formulations of the optimization problem are presented in Section 5, while the simulation and optimization results are presented in Section 6. Additionally, we compare the device-level and physical-level schematizations in terms of computational cost and accuracy: the performance of the rapidly prototyped layouts at device level are assessed through simulations at the physical level. Finally, Section 7 summarizes the main findings of the work and draws related conclusions.

## 2. Introduction to *feMEMSlite*

**Figure 1:**
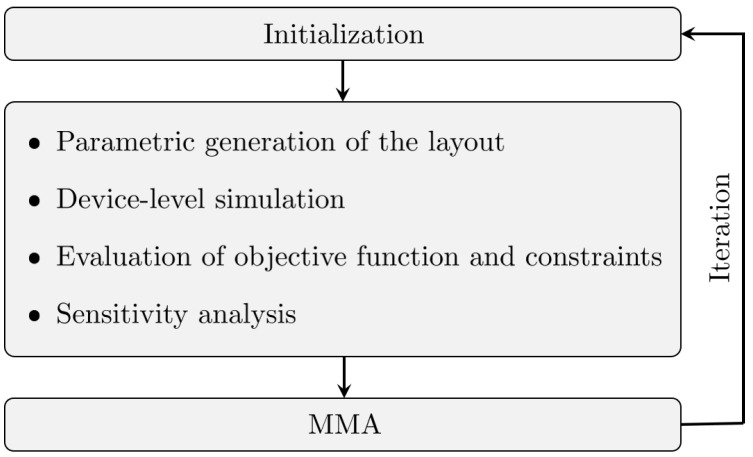
Workflow of *feMEMSlite*.

An overview of *feMEMSlite* is provided in Figure 1, along with the main iterative steps followed by the automatic design procedure. The general conceptual scheme of *feMEMSlite* is similar to *feMEMS* presented in [29], and the main differences are given by the use of a more efficient and more flexible schematization of the MEMS structure at device level.

The software requires as input a parametrization of the MEMS geometry by the design engineer, with design variables that are typically related to the position and dimensions of the composing mechanical elements, such as proof masses and springs. The structural layout is then automatically generated, and the linear dynamic behavior of the MEMS structure is simulated through a device-level schematization. This treats the proof masses as rigid bodies, discretizes the springs by Timoshenko beam finite elements, and accounts for electrostatic softening effects due to electromechanical interactions with additional negative spring constants (cf. Section 4.1). The automatic design of the structure follows a formulated optimization problem, whose objective function and constraints are computed from the results of the simulation. The optimization problem is solved using the gradient-based Method of Moving Asymptotes (MMA) [31], where the sensitivity analysis is performed by finite differences. Starting from an initial guess, the MMA allows one to iteratively update the MEMS layout until convergence. It is important to note that, in most cases, complex design problems are non-convex by nature. Therefore, gradient-based algorithms will converge to a local optimum that, in general, depends on the initial set of design variables. One way to better approximate the global optimum can be to increase the exploration of the design space by carrying out multiple optimizations starting with different initial guesses.

In order to allow a straightforward comparison between *feMEMS* and *feMEMSlite*, in the following we will employ the same reference design case and the same optimization objectives as in [29]: both the aspects will be recalled with proper references.

## 3. Reference Design Case and Associated Design Variables

**Figure 2:**
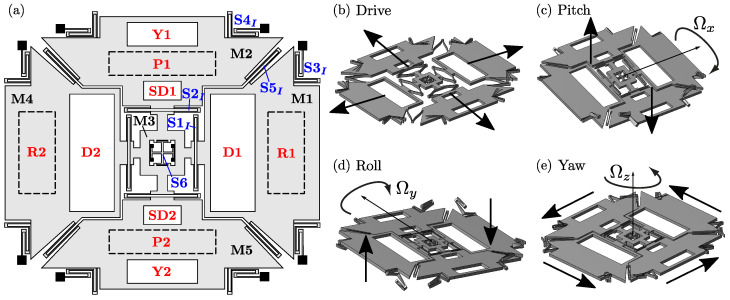
Reference layout of a triaxial beating heart MEMS gyroscope (**a**) and associated modal shapes: drive (**b**), sense pitch (**c**), sense roll (**d**), and sense yaw (**e**) modes. Redrawn from [29].

The details of the optimization steps are described considering the reference design case of a triaxial ”beating heart” MEMS gyroscope, shown in Figure 2, and considered also in [29]. In this section, after presenting the reference layout, we discuss the main associated design requirements and list the considered design variables.

### 3.1. Reference Triaxial Beating Heart MEMS Gyroscope Layout and Design Requirements

Triaxial beating heart MEMS gyroscopes, introduced by STMicroelectronics [5,32], are particularly relevant from the industrial point of view, given their ability to simultaneously detect angular rates along all three Cartesian axes, employing with one single internal microstructure. Referring to Figure 2a, they employ four proof masses, i.e., the ”roll” masses M1 and M4, and the ”pitch” masses M2 and M5. The proof masses are connected to the fixed anchor points (black squares) and coupled together, by means of different sets of folded springs and a central auxiliary cross mass (M3).

Two sets of comb finger electrodes [2] (drive: D1 and D2), located inside the roll masses, force the “drive” mode of the structure, consisting of the four proof masses moving closer to and away from the center of the device (Figure 2b). Two additional smaller sets of comb finger electrodes (sense of drive: SD1 and SD2), located here inside the pitch masses, are employed to differentially detect the drive motion, in order to achieve a closed-loop actuation and therefore a harmonic drive motion at resonance.

When the driven device is subjected to an external angular rate, sinusoidal Coriolis forces arise and excite the “sense” modes of the structure. Each sense mode is excited by a specific Cartesian component of the external angular rate: the yaw mode (Figure 2e) is forced by $\Omega_{z}$ and consists of an in-plane rotation of the structure, while the pitch and roll modes (Figure 2c,d) are forced by $\Omega_{x}$ and $\Omega_{y}$ and correspond to out-of-plane anti-phase motions of the opposing proof masses. The generated harmonic sense displacements are detected by variable-gap sensing electrodes [2] in a differential configuration and can be used as an indirect measure of the external angular rate. Proper variable-gap electrodes are therefore placed to detect the yaw (Y1 and Y2), roll (R1, R2), and pitch (P1, P2) displacements.

In order to obtain a suitable sensor performance, a careful choice of the structural natural frequencies has to be made. The value of the drive natural frequency $\omega_{d}$ usually depends on the features of the electronic driving circuit and on possible interactions with other close MEMS devices (e.g., in Inertial Measurement Units) and is generally set in the 10–30 kHz range [2]. In order to optimize the trade-off between a good sense response to the angular rate and a good robustness to variations in resonant frequencies or damping, the sense natural frequencies $\omega_{s}$ are usually kept at a 2–10% frequency mismatch with respect to the drive one [2]. In addition, aiming at achieving an adequately stiff enough structure along directions other than the drive and the sense ones, all the remaining “spurious” modes are usually kept at the highest possible frequencies.

### 3.2. Design Variables and Automatic Generation of the MEMS Geometry

The complexity of inertial MEMS structures such as beating heart MEMS gyroscopes, along with the variety of requirements to be satisfied, motivate the benefits of developing automatic design tools based on structural optimization. In order to do so, the MEMS structure layout is parametrized by a set of design variables, which are then optimized to maximize the performance of the device. Similarly to [29], and referring again to Figure 2a, the MEMS structure is first subdivided into its constituting mechanical elements, namely masses (denoted by “M”) and springs (denoted by “S”). The design variables are then used to describe the position and dimensions of these mechanical elements.

**Figure 3:**
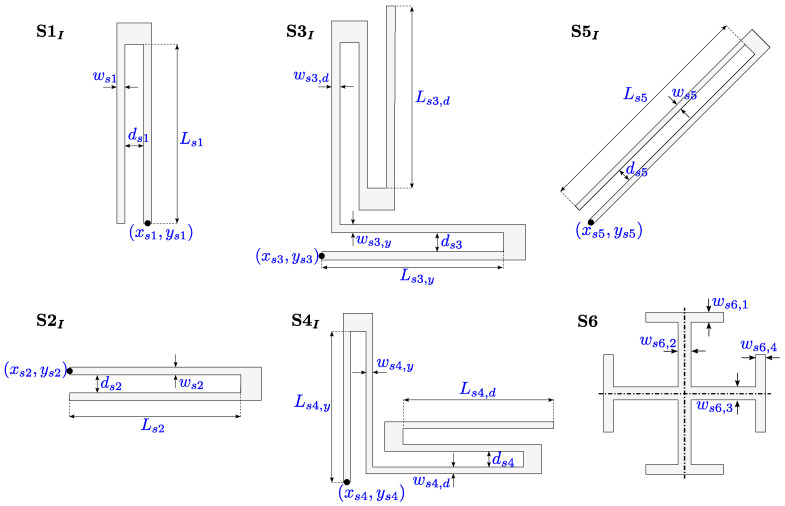
Design variables related to the geometry of the springs (redrawn from [29]).

Figure 3 shows the 33 design variables related to the springs: they are described by the (x,y) coordinates of an initial point and by parameters related to the geometry of the composing beams (such as width *w*, length *L*, and distance between parallel beams *d*). In particular, springs of similar type (e.g., U-shaped springs with different orientation $S1_{I}$, $S2_{I}$, $S5_{I}$ or serpentine springs with equivalent folds $S3_{I}$, $S4_{I}$) share the same parametrization and involve equivalent design parameters.

**Figure 4:**
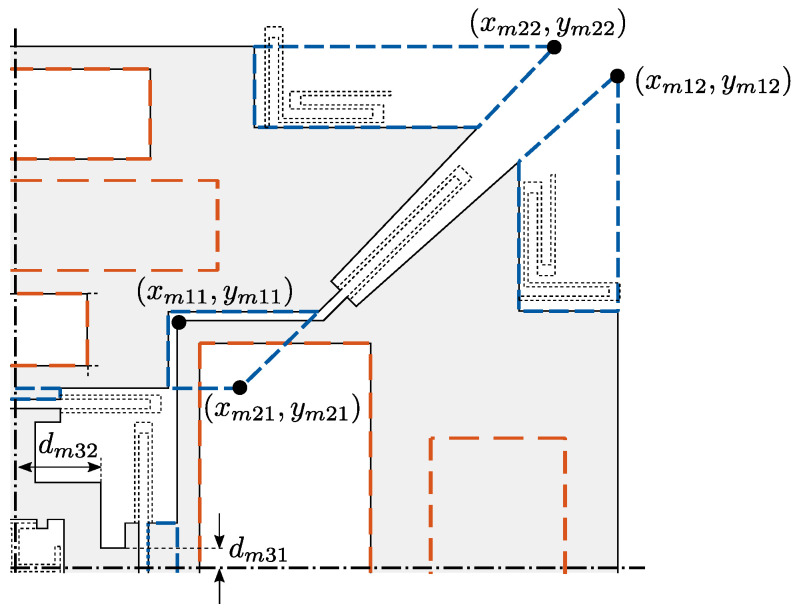
Design variables related to the geometry of the proof masses, removal of overlaps/disconnections in the layout (blue dashed lines), and generation of the space for the electrodes (green dashed lines). Before mirroring, the electrodes have the following dimensions related to one quarter of the structure and are kept at 25 μm from the borders of the proof masses (cf. nomenclature in Figure 2). D1: 190 μm × 250 μm, SD1: 80 μm × 80 μm, P1: 225 μm × 100 μm, R1: 150 μm × 150 μm, Y1: 150 μm × 100 μm. Redrawn from [29].

The 10 additional design variables related to the masses are shown in Figure 4: the roll and pitch masses M1 and M2 are initially described as trapezoidal shapes characterized by the $\left(x_{m},y_{m}\right)$ coordinates of four significant points. Such trapezoids also include local modifications to ensure sufficient room for electrode D1 and feasible connections with spring $S5_{I}$. The parametrization of the central cross-shaped mass M3 includes only the two dimensions indicated by $d_{m}$, but its geometry depends also on the position of the springs $S1_{I}$ and $S2_{I}$, to which it is kept connected.

The aforementioned parametrization leads to a set γ of 43 parameters used to describe the in-plane geometry of the MEMS device, wchih has a t=24μm out-of-plane thickness. In particular, by exploiting the symmetry of the layout, it is possible to parametrize only the first quadrant of the structure (subscript I), and then achieve the full layout through successive mirroring. We note that the choice of the design variables that compose the set γ is arbitrary, as long as it uniquely describes the design problem. Even if alternative formulations were possible, the one shown here has proven to be effective in obtaining well-performing devices for the presented design problems.

In the current version of the software, the element layouts are given as input to the design tool by manually listing the parametrized coordinates of nodal points for the folded springs and of vertices for the mass polygons. Future extensions of the software that make use of higher levels of abstractions (e.g., libraries of pre-parametrized standard mechanical elements) are certainly possible and are identified as future work.

As described in Figure 4, the algorithm finally performs some adjustments of the masses in order to remove residual overlaps/disconnections after the initial placement of the elements (blue dashed lines) and to create the needed space for in-plane and out-of-plane electrodes (red dashed lines). These adjustments are shown to be crucial in obtaining feasible geometries even for complex MEMS layouts and in fully exploiting the available footprint to automatically design adequately compact devices with optimized performance.

## 4. Device-Level Simulation of the MEMS Structure Behavior

### 4.1. Discretization of the Structure at the Device Level

**Figure 5:**
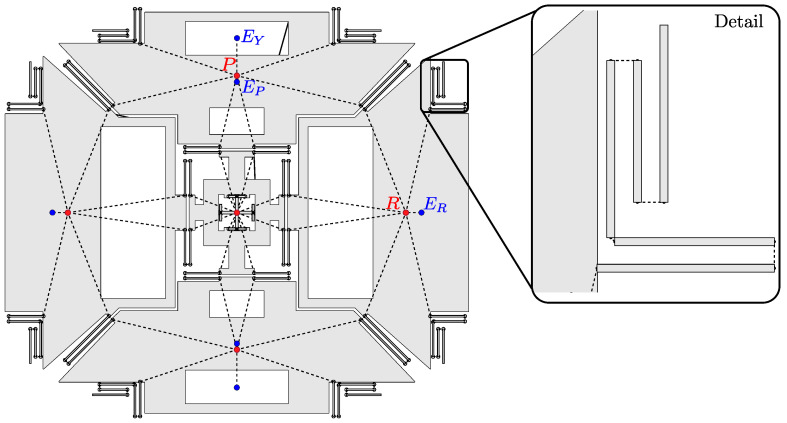
Device-level representation of the MEMS layout.

Once the geometry of the structure has been generated, the MEMS behavior is simulated using the schematization shown in Figure 5. The proof masses are schematized as rigid bodies whose inertial properties are condensed at their centers of gravity (red dots), while springs are discretized by Timoshenko beam finite elements (detail of Figure 5). Rigid connections (dashed lines) are imposed between the Timoshenko beams composing each spring, as well as between the springs and the masses they are attached to. This is done using Multi-Point Constraints (MPC, [33]), which impose a set of explicit kinematic constraints between the degrees of freedom of interest. One additional node is also considered at the center of each sense electrode (blue dots) and rigidly connected to the center of gravity of the corresponding proof mass. This allows one to properly compute the mechanical response of the sensor to the external input and also to account for electrostatic softening effects.

Each Timoshenko beam finite element [33] is associated with a mass matrix and a stiffness matrix. We therefore note that for the reference gyroscope design case, the contribution of the beams to the generated Coriolis forces is neglected.

The proof masses, modeled as rigid bodies, are instead only associated with mass properties; i.e., possible effects related to their flexibility are neglected. All the properties of the *i*-th rigid mass are associated with the degrees of freedom of its center of mass ${\left\{u_{x,mi},u_{y,mi},u_{z,mi},\theta_{x,mi},\theta_{y,mi},\theta_{z,mi}\right\}}^{T}$. Considering the *i*-th mass, the mass matrix $M_{i}$ accounting for the inertial properties, and the Coriolis matrix $G_{C,i}$, accounting for Coriolis forces in the gyroscope, are written as:

(1) $$\begin{array}{cc} M_{i}= & \left[\begin{array}{ccccccc} \; & m_{i} & 0 & 0 & 0 & 0 & 0\\ \; & 0 & m_{i} & 0 & 0 & 0 & 0\\ \; & 0 & 0 & m_{i} & 0 & 0 & 0\\ \; & 0 & 0 & 0 & J_{xx,i} & J_{xy,i} & J_{xz,i}\\ \; & 0 & 0 & 0 & J_{xy,i} & J_{yy,i} & J_{yz,i}\\ \; & 0 & 0 & 0 & J_{xz,i} & J_{yz,i} & J_{zz,i} \end{array}\right]\\ G_{C,i}\left(\Omega \right)=2m_{i} & \left[\begin{array}{ccccccc} \; & 0 & -\Omega_{z} & \Omega_{y} & 0 & 0 & 0\\ \; & \Omega_{z} & 0 & -\Omega_{x} & 0 & 0 & 0\\ \; & -\Omega_{y} & \Omega_{x} & 0 & 0 & 0 & 0\\ \; & 0 & 0 & 0 & 0 & 0 & 0\\ \; & 0 & 0 & 0 & 0 & 0 & 0\\ \; & 0 & 0 & 0 & 0 & 0 & 0 \end{array}\right] \end{array}$$

where $m_{i}$ is the total mass of the *i*-th rigid mass and $J_{xx,i}$, $J_{yy,i}$, $J_{zz,i}$, $J_{xy,i}$, $J_{xz,i}$, and $J_{yz,i}$ are its moments of inertia computed with respect to the three Cartesian axes. The skew-symmetric Coriolis matrix accounts for the Coriolis forces generated in the presence of an external angular rate $\Omega ={\left\{\Omega_{x}\;\Omega_{y}\;\Omega_{z}\right\}}^{T}$. Since for the considered design case the proof masses undergo a pure translation in the drive mode, we have neglected possible gyroscopic effects coupling rotational degrees of freedom in the definition of $G_{C,i}$.

The matrices related to rigid masses and Timoshenko beams are computed considering polysilicon material properties, i.e., a mass density $\rho =2330\;{\mathrm{kg}/m}^{3}$, a Young modulus E=148GPa, and a Poisson ratio ν=0.23.

A typical detection strategy for sense displacements in inertial MEMS devices is to use variable-gap parallel plate electrodes [1,2]. Due to the voltage difference between the proof mass and the electrode plate, electrostatic forces arise between the two. Furthermore, since the attractive electrostatic force increases when the plates gap decreases, this results in a negative equivalent spring constant, which reduces the overall stiffness of the electromechanical system and changes its natural sense frequencies. This effect, referred to as electrostatic softening, can have an important impact, e.g., on the drive-sense frequency mismatch of the gyroscope. In the following paragraphs, the derivation of the negative spring constants associated with out-of-plane (pitch, roll) and in-plane (yaw) variable-gap detection electrodes is presented.

#### 4.1.1. Out-of-Plane Variable-Gap Capacitance

**Figure 6:**
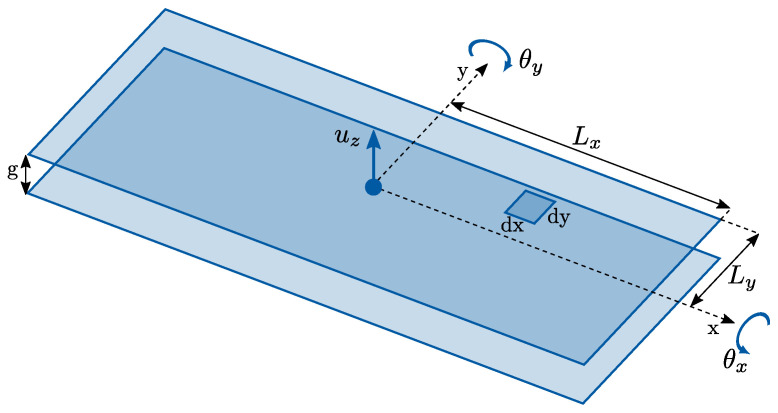
Scheme of an out-of-plane capacitor as a function of $u_{z}$, $\theta_{x}$, $\theta_{y}$.

In order to detect the out-of-plane motion of proof masses (e.g., due to pitch and roll angular rates), rectangular detection electrodes are usually placed beneath the proof masses, resulting in out-of-plane variable-gap capacitors as shown in Figure 6. Here, the top plate (free to move) is represented by the proof mass bottom surface, while the bottom plate (fixed) is represented by the electrode on the substrate. The overall capacitance is a function of the vertical displacement $u_{z}$ of the top plate and of its out-of-plane rotations $\theta_{x}$ and $\theta_{y}$. In particular, the infinitesimal capacitance in a generic position (x,y) is

(2) $$\begin{array}{c} dC_{oop}=\varepsilon_{0}\frac{dxdy}{g+u_{z}+\theta_{x}y-\theta_{y}x}=\frac{\varepsilon_{0}}{g}{\left(1+\frac{u_{z}}{g}+\frac{\theta_{x}y}{g}-\frac{\theta_{y}x}{g}\right)}^{-1}dxdy \end{array}$$

where *g* is the initial gap between the two plates and $\varepsilon_{0}=8.854\times 10^{-12}$ F/m is the vacuum electric permittivity. Recalling the following Taylor expansion:

(3) $${\left(1+x+y+z\right)}^{-1}\approx 1-x-y-z+x^{2}+y^{2}+z^{2}+2xy+2xz+2yz$$

we can write:

(4) $$\begin{array}{cc} dC_{oop}\approx \frac{\varepsilon_{0}}{g}\left(1-\frac{u_{z}}{g}-\frac{\theta_{x}y}{g}+\frac{\theta_{y}x}{g}+\frac{u_{z}^{2}}{g^{2}}+\frac{\theta_{x}^{2}y^{2}}{g^{2}}\right. & \\ \left.+\frac{\theta_{y}^{2}x^{2}}{g^{2}}+2\frac{u_{z}\theta_{x}y}{g^{2}}-2\frac{u_{z}\theta_{y}x}{g^{2}}-2\frac{\theta_{x}\theta_{y}xy}{g^{2}}\right) & dxdy \end{array}$$

The total out-of-plane capacitance as a function of $u_{z}$, $\theta_{x}$, $\theta_{y}$ can be then computed by integrating Equation (4) over the electrode area defined by the dimensions $L_{x}$ and $L_{y}$:

(5) $$\begin{array}{cc} C_{oop} & =\int_{-L_{x}}^{L_{x}}\int_{-L_{y}}^{L_{y}}dC_{oop}\\ & =\frac{\varepsilon_{0}}{g}\left(4L_{x}L_{y}-4\frac{L_{x}L_{y}}{g}u_{z}+4\frac{L_{x}L_{y}}{g^{2}}u_{z}^{2}+\frac{4}{3}\frac{L_{x}L_{y}^{3}}{g^{2}}\theta_{x}^{2}+\frac{4}{3}\frac{L_{x}^{3}L_{y}}{g^{2}}\theta_{y}^{2}\right) \end{array}$$

#### 4.1.2. In-Plane Variable-Gap Capacitance

**Figure 7:**
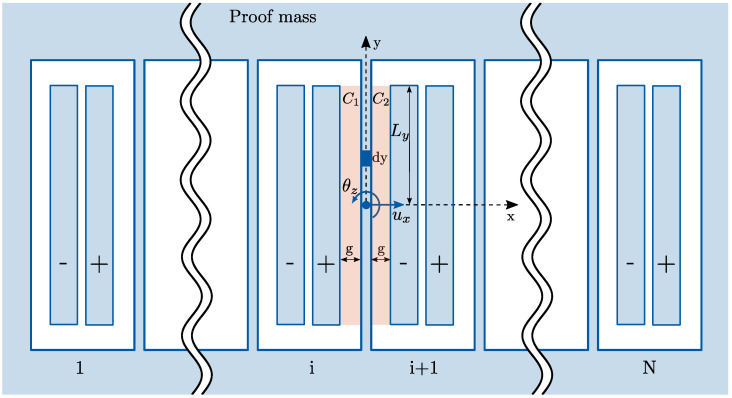
Scheme of an in-plane capacitor as a function of $u_{x}$, $\theta_{z}$.

The in-plane motion of the proof masses is detected by a series of differential in-plane electrodes as shown in Figure 7. The represented configuration allows one to detect displacements along the *x* direction, and differential sensing is obtained through two sets of capacitors that are oppositely influenced by proof mass displacements, namely $C_{1}$ between the proof mass and the fixed plates (“+”) and $C_{2}$ between the proof mass and the fixed plates (“−”).

In order to compute the total capacitance as a function of the displacement $u_{x}$ and the rotation $\theta_{z}$, we first write the expressions of the infinitesimal capacitance value at a generic coordinate *y* as:

(6) $$dC_{1}=\frac{\varepsilon_{0}t}{g}{\left(1+\frac{u_{x}}{g}-\frac{\theta_{z}y}{g}\right)}^{-1}dy\approx \frac{\varepsilon_{0}t}{g}\left(1-\frac{u_{x}}{g}+\frac{\theta_{z}y}{g}+\frac{u_{x}^{2}}{g^{2}}+\frac{\theta_{z}^{2}y^{2}}{g^{2}}-2\frac{u_{x}\theta_{z}y}{g^{2}}\right)dy$$

(7) $$dC_{2}=\frac{\varepsilon_{0}t}{g}{\left(1-\frac{u_{x}}{g}+\frac{\theta_{z}y}{g}\right)}^{-1}dy\approx \frac{\varepsilon_{0}t}{g}\left(1+\frac{u_{x}}{g}-\frac{\theta_{z}y}{g}+\frac{u_{x}^{2}}{g^{2}}+\frac{\theta_{z}^{2}y^{2}}{g^{2}}-2\frac{u_{x}\theta_{z}y}{g^{2}}\right)dy$$

where *t* is the out-of-plane thickness of the MEMS structure. The total in-plane capacitance can be then computed by integrating Equations (6) and (7) along the length of the capacitance characterized by the dimension $L_{y}=40$μm and by considering a number $N_{ip}=12$ of differential electrodes:

(8) $$C_{ip}=N_{ip}\left(\int_{-L_{y}}^{L_{y}}dC_{1}+\int_{-L_{y}}^{L_{y}}dC_{2}\right)=N_{ip}\frac{\varepsilon_{0}t}{g}\left(4L_{y}+4\frac{L_{y}}{g^{2}}u_{x}^{2}+\frac{4}{3}\frac{L_{y}^{3}}{g^{2}}\theta_{z}^{2}\right)$$

#### 4.1.3. Electrostatic Softening Effects

In order to study the electrostatic softening effects, we first write the expression of the energy stored by the out-of-plane and in-plane capacitors:

(9) $$U_{oop}=\frac{1}{2}C_{oop}V^{2},\;U_{ip}=\frac{1}{2}C_{ip}V^{2}$$

where *V* is the voltage difference between the proof mass and the fixed plates of the capacitors. The equivalent electrostatic stiffness matrices can be computed as [1,2]:

(10) Kes,oop=−∂2Uoop∂xixj=−12∂2Coop∂xixjV2=−diag4ε0LxLyg3V2,43ε0LxLy3g3V2,43ε0Lx3Lyg3V2Kes,ipp=−∂2Uip∂xixj=−12∂2Cip∂xixjV2=−Nipdiag4ε0Lytg3V2,43ε0Ly3tg3V2

where $x_{i}$, $x_{j}$ indicate the degrees of freedom influencing the capacitance, namely $u_{z}$, $\theta_{x}$, $\theta_{y}$ for out-of-plane electrodes and $u_{x}$, $\theta_{z}$ for in-plane electrodes. We remark that the electrostatic spring constants in Equation (10) are negative, and they therefore result in a reduction in the natural frequencies of the structure. In particular, an initial electrode gap g=2μm and a voltage difference V=10 V are considered below.

### 4.2. Equations of Motion and Dynamic Analyses

After assembling the rigid proof masses, Timoshenko beams and electrostatic negative spring constants, the equations of motion of the MEMS structure can be written in the form:

(11) $$M\ddot{u}+\left(C+G_{C}\left(\Omega \right)\right)\dot{u}+Ku=f$$

where u and f are the vectors of degrees of freedom and loads, M and K are the mass and the stiffness matrices, $G_{C}\left(\Omega \right)$ is the skew-symmetric Coriolis matrix, and C is a suitable damping matrix. In particular, since the dimensionless damping ratio in MEMS gyroscopes is usually $∼10^{-3}$ [2], for the considered example, we will neglect the effect of damping, also because an adequate drive-sense mismatch will be required in the formulation of the optimization problem [2] (cf. Section 5).

One important goal in the design of MEMS devices is to achieve appropriate modal behavior of the structure. Starting from Equation (11), the following eigenvalue problem is used to obtain the first $n_{\omega }$ undamped natural frequencies $\omega_{j}$ and the related mode shapes $\Phi_{j}$:

(12) $$\left(-\omega_{j}^{2}M+K\right)\Phi_{j}=0\;,\;j=1,\cdots ,n_{\omega }$$

Equation (12) allows to identify the natural frequencies of interest, e.g., for MEMS gyroscopes the drive natural frequency and mode shape $\left(\omega_{d},\Phi_{d}\right)$, as well as the three sense natural frequencies and the related mode shapes $\left(\omega_{n},\Phi_{n}\right),\;n=x,y,z$, i.e., pitch, roll and yaw.

Further design requirements can apply to the modal shapes associated with the natural frequencies of interest. For example, in the case of beating-heart MEMS gyroscopes, it is general desirable to achieve a similar drive motion between the different proof masses, especially when the drive and drive detection electrodes are not place on the same mass. This enhances the robustness of the closed loop excitation and the possibility to achieve adequate responses to the different Cartesian components of the angular rate. It is therefore useful to define a drive-mode shape parameter $\delta_{d}$ [29], as the ratio between the displacements $u_{y,P}$ and $u_{x,R}$ of the pitch and roll centers of mass (points P and R defined in Figure 5), in the drive mode shape $\Phi_{d}$:

(13) $$\delta_{d}=\frac{u_{y,P}}{u_{x,R}}=\frac{{l_{d,p}}^{T}\Phi_{d}}{{l_{d,r}}^{T}\Phi_{d}}$$

where $l_{d,p}$ and $l_{d,r}$ are appropriate extraction vectors. The objective will therefore be to keep $\delta_{d}$ defined in Equation (13) as close as possible to 1.

Finally, the main interest of the design is to maximize the sensor response to the external input. For the reference example, we calculate the mechanical response of the gyroscope to the different Cartesian components of the external angular rate, performing the following three harmonic response analyses [29]:

(14) $$\begin{array}{cc} \; & \left(-\omega_{h,n}^{2}M+i\omega_{h,n}G_{C}\left(\Omega_{n}\right)+K\right)u_{h,n}=f_{h,n}\\ \; & \omega_{h,n}=\alpha_{s}\omega_{n},\;n=x,y,z \end{array}$$

where $u_{h,n}$ and $f_{h,n}$ are the vectors of harmonic displacements and harmonic loads, whereas $\omega_{h,n}$ are the frequencies of excitation. In particular, we consider test angular rates with amplitude 300°/s, along the pitch ($\Omega_{x}={\left\{300{\;}^{{}^{\circ}}/s,0,0\right\}}^{T}$), roll ($\Omega_{y}={\left\{0,300{\;}^{{}^{\circ}}/s,0\right\}}^{T}$), and yaw ($\Omega_{z}={\left\{0,0,300{\;}^{{}^{\circ}}/s\right\}}^{T}$) axes. Furthermore, in the analyses we impose a harmonic 5 μm drive displacement to the $u_{y,P}$ and $u_{x,R}$ degrees of freedom at frequencies $\omega_{h,n}=\alpha_{s}\omega_{n}$, where $\alpha_{s}=0.95$ is used to simulate an operating frequency that is 5% lower than the sense natural frequencies. The focus of the analyses is on the Coriolis displacements detected at the centers of the sense electrodes (points $E_{R}$, $E_{P}$, $E_{Y}$ in Figure 5), i.e., $u_{Cor,x}=\left|u_{z,E_{P}}\right|$, $u_{Cor,y}=\left|u_{z,E_{R}}\right|$, $u_{Cor,z}=\left|u_{x,E_{Y}}\right|$, which can be extracted from the solution of Equation (14) using appropriate extraction vectors $l_{h,n}$:

(15) $$u_{Cor,n}=\left|{l_{h,n}}^{T}u_{h,n}\right|,\;n=x,y,z$$

*feMEMSlite* software is fully developed in MATLAB: for what concerns the needed numerical solvers, the eigenvalue problem to compute the natural frequencies (Equation (12)) is solved with the eigs function, whereas the linear systems to compute the harmonic responses (Equation (14)) are solved by UMFPACK (function “∖”).

## 5. Definition of the Optimization Problem

### 5.1. Proposed Formulations

The formulation of the optimization problem translates into mathematical terms the design objectives: usually the aim is to maximize the sensor performance or response while satisfying a set of constraints, e.g., related to the modal behavior of the structure. For what concerns the reference gyroscope design case, two possible formulations problem are presented, similar to the ones treated in [29].

The first optimization problem (P1) aims to maximize the response to the external angular rates while matching given target natural frequencies and their desired ordering:

(16) $$\begin{array}{cccccc} \; & \max_{\gamma }\; & \; & \min(u_{Cor,n})\\ \; & s.t.\; & \; & |\omega_{d}-\omega_{d,des}|\leq \varepsilon_{d}\\ \; & \; & & |\omega_{d}/\omega_{n}-\alpha_{s}|\leq \varepsilon_{s},\; & \; & \mathrm{for}\;n=x,y,z\\ \; & \; & & \omega_{sp,1}\geq \omega_{n}+\Delta \omega_{sp,min},\; & \; & \mathrm{for}\;n=x,y,z\\ \; & \; & & |\delta_{d}-1|\leq \varepsilon_{\delta }\\ \; & \; & & \mathrm{Distance}\;\mathrm{constraints} \end{array}$$

The second optimization problem (P2) is a simplification of (P1) and is mainly focused on the tuning of the structural eigenfrequencies. In particular, we aim at maximizing the distance of the spurious modes from the operating frequency range while matching the target drive and sense natural frequencies:

(17) $$\begin{array}{cccccc} \; & \max_{\gamma }\; & \; & \omega_{sp,1}-\omega_{d}\\ \; & s.t.\; & \; & |\omega_{d}-\omega_{d,des}|\leq \varepsilon_{d}\\ \; & \; & & |\omega_{d}/\omega_{n}-\alpha_{s}|\leq \varepsilon_{s},\; & \; & \mathrm{for}\;n=x,y,z\\ \; & \; & & |\delta_{d}-1|\leq \varepsilon_{\delta }\\ \; & \; & & \mathrm{Distance}\;\mathrm{constraints} \end{array}$$

In (P1), we maximize the minimum response to the considered angular rates along the three Cartesian axes, while in (P2), we maximize the distance between the first spurious and the drive natural frequency, i.e., $\omega_{sp,1}$ and $\omega_{d}$. In both formulations, constraints are imposed to match the target drive natural frequency $\omega_{d,des}$ with a tolerance $\varepsilon_{d}=100$ Hz, and to achieve a 5% mismatch between the drive and the sense natural frequencies, by prescribing that the ratio $\omega_{d}/\omega_{n}$ matches the value $\alpha_{s}=0.95$ with a tolerance $\varepsilon_{s}=0.0025$. Additionally, in (P1), the distance between the first spurious mode and the sense natural frequencies must be greater than $\Delta \omega_{sp,min}=3000$ Hz. The drive mode shape parameter $\delta_{d}$ (Equation (13)) is in both cases prescribed to match the desired value of 1 with a tolerance $\varepsilon_{\delta }=0.01$, while ”Distance constraints” are imposed not to exceed the considered 700 μm × 600 μm design space and to impose a minimum distance of 25 μm between the the different elements, such as electrodes and springs. We finally note that proper normalizations of objective functions and constraints can be introduced to adequately improve the convergence of the MMA, following the directions provided in [34], as done in [29]. Normalization terms have not been included in Equations (16) and (17) for the sake of clarity in the interpretation of the formulations, and the reader is pointed to the aforementioned references for more details.

### 5.2. Sensitivity Analysis

The gradient based Method of Moving Asymptotes (MMA) [31] is used to solve the formulated optimization problems. The sensitivities of the objective function and constraints with respect to changes in the design variables can be computed with different methods [35].

Adjoint sensitivity analysis is usually employed for computationally expensive simulations, such as physical-level ones (e.g., in [29]). The main advantages of adjoint sensitivity analysis are related to its accuracy and its numerical efficiency: its computational cost scales linearly with the number of functions to be differentiated, which for industrially relevant design cases is usually lower than the number of design variables. However, formulating and solving the related adjoint problems can be cumbersome, and the integration with the simulation code is, at times, challenging.

For the reasons highlighted above, we propose a finite differences method, which simply involves perturbing each design variable and performing an additional simulation each time. While this obviously requires an additional computational effort (which scales linearly with the number of design variables), device-level simulations are fairly cheap, such that the overall computational cost is contained. This allows us to obtain an extremely flexible tool that only requires minimal human intervention and effort whenever new problem formulations are introduced.

## 6. Results and Discussion

In this Section, the simulation and optimization results obtained with *feMEMSlite* are discussed. First, the proposed device-level schematization is validated with a comparison with the physical-level schematization from *feMEMS* and Abaqus commercial software (taken as reference), focusing on accuracy and computational costs. Second, the layouts optimized with *feMEMSlite* are presented, following the two formulations of the problem described in Section 5. Finally, we validate the rapidly prototyped layouts at device-level with physical-level simulations.

### 6.1. Validation of the Proposed Device-Level Schematization

In the following, the accuracy and computational cost of *feMEMSlite* device-level schematization are discussed. This is done by comparing with the physical-level simulations from *feMEMS* and Abaqus, whose results reported in Table 1 and Table 2 are taken from [29]. We recall that *feMEMS* employs quadratic hexaedral elements to mesh the springs and quadratic triangular shell elements to mesh the proof masses, while in Abaqus, the structure is fully discretized by quadratic hexedral elements (HEX20). All the results refer to the example gyroscope layout shown in Figure 2a and are obtained on a Intel Core i7-6800K CPU (3.40 GHz).

Referring to Table 1, it can be seen how the proposed device-level schematization in *feMEMSlite* guarantees an extremely low computational cost. For what concerns the time taken by the simulation, i.e., the time needed to assemble the matrices of the system and to perform the modal and the harmonic response analyses, we achieve an improvement by three orders of magnitude with respect to Abaqus and one of two orders of magnitude lower with respect to *feMEMS*. A further considerable time reduction with respect to usual commercial FEM softwares is given in *feMEMSlite* and *feMEMS* by the automatic generation of the geometric layout: this improvement is less easy to explicitly quantify and results are user-dependent, but we estimate that it can be in the order of some of minutes.

Table 2 shows how the high reduction in computational cost of *feMEMSlite* is associated with a certain degree of approximation with respect to Abaqus, which is increased with respect to *feMEMS*. In particular, for purely mechanical simulations, 2–5% of errors are related to the eigenfrequencies and the harmonic response for in-plane motion, while errors around 11–13% are associated with out-of-plane motion. Furthermore, the last column in Table 2 shows the effect of adding electrostatic softening in device-level simulations: the sense eigenfrequencies of the structure are slightly reduced, while the drive eigenfrequency is not influenced.

The analysis of computational costs and accuracy already underlines the potentialities of *feMEMSlite* in allowing the fast design of MEMS devices, provided that the increased degree of approximation does not affect the effectiveness of the optimized layouts. In the following, we show some examples of rapidly prototyped gyroscope layouts at a device level, and we assess their performance when moving to physical-level design.

### 6.2. Optimization Results

**Figure 8:**
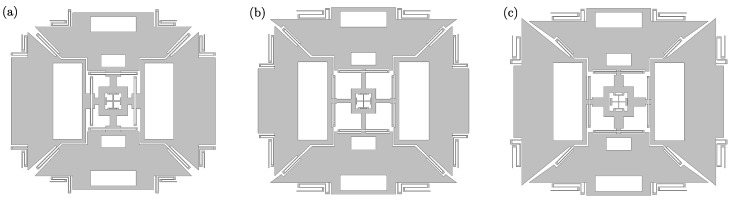
(**a**) Initial layout guess, (**b**) layout optimized by solving (P1), (**c**) layout optimized by solving (P2).

**Figure 9:**
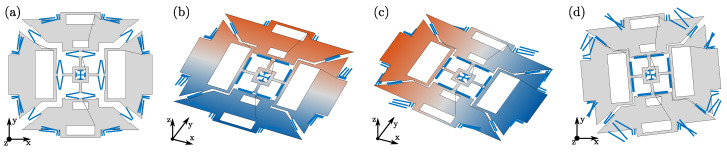
Modal shapes of the layout optimized by solving (P1) (Figure 8b): drive (**a**), pitch (**b**), roll (**c**), and yaw (**d**).

**Figure 10:**
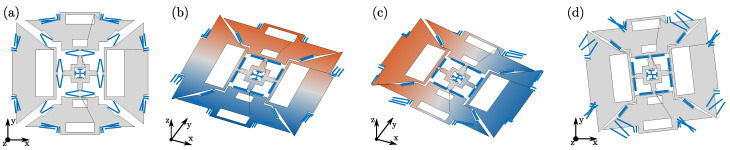
Modal shapes of the layout optimized by solving (P2) (Figure 8c): drive (**a**), pitch (**b**), roll (**c**), and yaw (**d**).

The optimized layouts obtained by solving problems (P1) and (P2) are shown in Figure 8b,c, respectively. Figure 8a represents instead the initial guess, that is, the reference layout from Figure 2a already used in the previous sections to illustrate the features of the proposed design tool. Table 3 compares the natural frequencies, the drive mode shape parameter and the Coriolis sense displacements between the different layouts. The drive and sense mode shapes of layouts (b) and (c) are shown in Figure 9 and Figure 10, where a target drive frequency $f_{d}=\omega_{d}/2\pi =20$ kHz is considered.

Table 3 shows how the optimizer is able to converge correctly for both problems, even though the chosen starting point is infeasible for the considered example. The initial layout guess (a) in fact does not respect the desired order of the natural frequencies: several spurious modes appear between the drive and roll modes, and the yaw natural frequency is below the drive one. Furthermore, the pitch mode is not among the first eight modes, and the target drive natural frequency is missed by more than 14 kHz. However, referring again to Table 3, both the optimized layouts (b) and (c) fully respect the requirements on the target natural frequencies and their ordering. The drive modes (Figure 9a and Figure 10a) are placed at the 20 kHz target frequency, and the sense modes (Figure 9b–d and Figure 10b–d) are at the target 5% mismatch. In particular, it is possible to see how the optimizer increases the compliance of the suspending and coupling springs by increasing the length of the composing beams, with the aim of reducing the natural frequencies from the initial guess layout (a) to the optimized layouts (b) and (c) and therefore match the target values. Furthermore, for both layouts (b) and (c), similar displacements of the pitch and roll masses are kept in the drive mode shape, i.e., $\delta_{d}$ is maintained adequately close to 1.

In Table 3, we can also see how the algorithm is able to indeed maximize the objective functions for both problems (P1) and (P2). When the objective is to maximize the spurious frequencies ((P2): layout (c)), the first spurious mode is placed at a 3549 Hz mismatch from the sense modes. Instead, for layout (b), the first spurious mode is kept at the minimum possible distance $\Delta \omega_{sp,min}=3000$ Hz. When maximum sense displacements are the desired ((P1) layout (b)), a minimum Coriolis displacement of about 2.95 nm is obtained. We note that, because of the overall structural stiffness reduction during the optimization process, the minimum Coriolis displacement increases also for layout (c), where it is shown to be almost doubled compared to layout (a).

### 6.3. Simulation of the Optimized Layouts at Physical Level

The final aim of the discussion is to assess how the layouts optimized at device level with *feMEMSlite* perform when considering the physical-level design. This is done by comparing the simulation results for layouts (b) and (c) between *feMEMSlite* and Abaqus HEX20. The results are reported in Table 4.

The layouts optimized at the device level perform well even when considering physical-level schematizations. In fact, the drive frequency is also close to the 20 kHz target in Abaqus simulations, and the different natural frequencies maintain the desired mode ordering, with a suitable distance between the operating range and spurious modes. Moreover, the drive mode shape parameter $\delta_{d}$ is kept close to 1, and the Coriolis responses simulated at a physical level are close to their device-level estimations. Fine-tuning adjustments to precisely match the target drive natural frequency and the sense mismatch can be easily performed by the design engineer, possibly relying on physical-level optimizers (e.g., with *feMEMS* [29]).

## 7. Conclusions

In this paper, we have presented an automatic design tool for inertial MEMS devices—*feMEMSlite* —that employs a computationally cheap schematization at the device level. It allows a rapid device prototyping and represents a valid alternative to more sophisticated physical-level design tools.

The general workflow of *feMEMSlite* has been inherited from its physical-level counterpart *feMEMS*: the proposed tool allows the parametric and automatic generation of the MEMS geometry, the simulation of the device dynamics, and the optimization of the layout. In order to demonstrate the effectiveness of the proposed tool, we have presented the design example of triaxial beating heart MEMS gyroscopes, in which 43 design variables have been employed to describe the dimensions and position of its internal mechanical elements.

In *feMEMSlite*, the schematization of the MEMS structure is performed by treating the proof masses as rigid bodies, discretizing the flexural springs by Timoshenko beam finite elements, and considering additional negative spring constants to account for the electrostatic softening effects. The derivations of such spring constants, related to both translational and rotational degrees of freedom of the proof masses, are also presented.

The reference layout has been optimized following two possible industrially-relevant problems: achieving a sensor with the maximum response to the external angular rate and maximizing the distance of spurious natural frequencies from the operating frequency range. Appropriate constraints have been included to satisfy technological and performance requirements and to match the target eigenfrequencies of the structure. The formulated optimization problems have been solved using the gradient-based Method of Moving Asymptotes (MMA), and the needed sensitivity analysis has been performed through a finite differences scheme in order to maximize the tool flexibility.

Finally, the rapidly prototyped layouts at device level have been validated by comparing the results with those obtained running full 3D simulations in *feMEMS* and Abaqus. The efficient schematization of *feMEMSlite* reduces the simulation time by three orders of magnitude vs. Abaqus and by two orders of magnitude vs. *feMEMS*, with a general slight overestimation of the structural eigenfrequencies (around 5–10%). The layouts optimized at the device level therefore show good performances also when simulated at physical level: a correct mode ordering is maintained, while preserving adequate responses to the external angular rates and proper distance of spurious modes from the operational frequency range. Only minor adjustments are therefore required to fine-tune the design.

While the current version of the tool requires as manual input the list of parametrized nodal points for the springs and polygons for the masses, future versions can introduce libraries of pre-parametrized standard mechanical elements. In addition, the employed schematization can be extended to capture mechanical nonlinearities and multiphysical effects, such as nonlinear electrostatics, thermoelasticity and fluid–structure interactions.

## Figures and Tables

**Table 1:** Abaqus (HEX20), *feMEMS*, and *feMEMSlite*: run-time comparison. Data are partially taken from [29].

	Abaqus [29]	*feMEMS* [29]	*feMEMSlite*
**Geom. creation time**	(user dependent, $∼10^{2}$ s)	0.2 s	0.2 s
**Assembly time**	8.4 s	17.3 s	0.003 s
**Modal analysis time**	46.3 s	0.001 s	0.005 s
**Harm. analysis time**	89.4 s × 3	0.001 s × 3	0.005 s × 3
**Total time**	322.8 s	17.5 s	0.25 s
**Number of dofs**	672,765	416,089 (full), 246 (red.)	354

**Table 2:** Simulation results obtained using Abaqus (HEX20), *feMEMS*, and *feMEMSlite*. Data are partially taken from [29].

	Abaqus [29]	*feMEMS* [29]	*feMEMSlite* (no soft.)	*feMEMSlite* (+soft.)
$f_{\mathrm{drive}}$	31,975 Hz	31,931 Hz (−0.14%)	34,108 Hz (+6.67%)	34,108 Hz
$f_{\mathrm{pitch}}$	43,018 Hz	42,980 Hz (−0.09%)	47,780 Hz (+11.07%)	47,679 Hz
$f_{\mathrm{roll}}$	38,761 Hz	38,718 Hz (−0.11%)	43,187 Hz (+11.42%)	43,045 Hz
$f_{\mathrm{yaw}}$	30,796 Hz	30,772 Hz (−0.08%)	31,694 Hz (+2.92%)	31,605 Hz
$f_{\mathrm{sp},1}$	34,478 Hz	34,440 Hz (−0.11%)	35,775 Hz (+3.76%)	35,775 Hz
$\delta_{d}$	0.9866	0.9874 (+0.08%)	0.9922 (+0.57%)	0.9922
$u_{\mathrm{Cor},x}$	1.4136 nm	1.3954 nm (−1.29%)	1.2266 nm (−13.23%)	1.2295 nm
$u_{\mathrm{Cor},y}$	1.7863 nm	1.7648 nm (−1.20%)	1.5828 nm (−11.39%)	1.5885 nm
$u_{\mathrm{Cor},z}$	2.1082 nm	2.0534 nm (−2.60%)	2.0620 nm (−2.19%)	2.0970 nm

**Table 3:** Comparison of the different layouts shown in Figure 8 in terms of natural frequencies, drive mode shape parameter, and Coriolis displacements.

	Layout (a)	Layout (b)	Layout (c)
**Formulation**	- (*initial guess*)	(P1)	(P2)
$f_{1}$	31,605 Hz (yaw)	20,100 Hz (drive)	20,099 Hz (drive)
$f_{2}$	34,108 Hz (drive)	21,153 Hz (pitch)	21,139 Hz (pitch)
$f_{3}$	35,775 Hz (spurious)	21,213 Hz (yaw)	21,211 Hz (yaw)
$f_{4}$	36,312 Hz (spurious)	21,213 Hz (roll)	21,212 Hz (roll)
$f_{5}$	39,715 Hz (spurious)	24,213 Hz (spurious)	24,761 Hz (spurious)
$f_{6}$	43,045 Hz (roll)	24,214 Hz (spurious)	24,761 Hz (spurious)
$f_{7}$	46,259 Hz (spurious)	28,993 Hz (spurious)	24,770 Hz (spurious)
$f_{8}$	47,047 Hz (spurious)	30,064 Hz (spurious)	28,090 Hz (spurious)
$\delta_{d}$	0.9922	1.010	0.9949
$\omega_{d}/\omega_{p}$	0.7154	0.9525	0.9502
$\omega_{d}/\omega_{r}$	0.7924	0.9525	0.9475
$\omega_{d}/\omega_{y}$	1.0792	0.9525	0.9475
$u_{\mathrm{Cor},x}$	1.2295 nm	2.9509 nm	2.3950 nm
$u_{\mathrm{Cor},y}$	1.5885 nm	2.9509 nm	2.9725 nm
$u_{\mathrm{Cor},z}$	2.0970 nm	3.5064 nm	3.1257 nm

**Table 4:** Validation of the optimized layouts: comparison with Abaqus HEX20.

Layout	(b) *feMEMSlite*	(b) Abaqus HEX20	(c) *feMEMSlite*	(c) Abaqus HEX20
$f_{1}$	20,100 Hz (drive)	19,416 Hz (drive)	20,099 Hz (drive)	19,383 Hz (drive)
$f_{2}$	21,153 Hz (pitch)	19,912 Hz (pitch)	21,139 Hz (pitch)	19,581 Hz (pitch)
$f_{3}$	21,213 Hz (yaw)	20,125 Hz (roll)	21,211 Hz (yaw)	19,810 Hz (roll)
$f_{4}$	21,213 Hz (roll)	20,790 Hz (yaw)	21,212 Hz (roll)	20,837 Hz (yaw)
$f_{5}$	24,213 Hz (spur.)	23,677 Hz (spur.)	24,761 Hz (spur.)	23,964 (spur.)
$\delta_{d}$	0.9922	1.0090	0.9949	0.9927
$u_{\mathrm{Cor},x}$	2.9509 nm	3.0498 nm	2.3950 nm	2.5316 nm
$u_{\mathrm{Cor},y}$	2.9509 nm	3.0630 nm	2.9725 nm	2.9406 nm
$u_{\mathrm{Cor},z}$	3.5064 nm	3.4003 nm	3.1257 nm	3.0543 nm

## Data Availability

Confidentiality restrictions apply to the availability of the software source code, as the present research work is part of a cooperation between Politecnico di Milano and STMicroelectronics. However, the manuscript provides all the relevant information needed by readers to replicate the presented results, and further material can be shared upon request to the corresponding author, with permission of STMicroelectronics.
